# Assessment of Gingival Health Status among 5- and 12-Year-Old Children in Yemen: A Cross-Sectional Study

**DOI:** 10.1155/2013/352621

**Published:** 2013-06-26

**Authors:** Khaled A. Al-Haddad, Yahia T. Ibrahim, Ahmed M. Al-Haddad, Nezar N. Al-Hebshi

**Affiliations:** ^1^Department of Orthodontic and Pediatric Dentistry, College of Dentistry, Sana'a University, Sana'a, Yemen; ^2^Department of Conservative, Khartoum University, Khartoum, Sudan; ^3^Department of Community Medicine, College of Medicine, Sana'a University, Sana'a, Yemen; ^4^Department of Periodontology, Faculty of Dentistry, University of Jazan, Saudi Arabia

## Abstract

*Purpose*. There are limited data about the gingival health status in Yemeni children. The aim, therefore, was to assess oral hygiene status and prevalence and severity of gingivitis among Yemeni preschool and school children. *Materials and Methods*. A total of 5396 children were included from 5 representative Yemeni governorates: Sana'a, Hajjah, Hodeida, Hadramaut, and Taiz. Five-year olds (1292) were recruited from private kindergartens while 12-year olds (4104) were selected from public primary schools. Gingival health status was assessed using the plaque index (PI), calculus index (CAI), and gingival index (GI) on the 6 Ramfjord teeth. The latter index was used to categorize gingivitis severity at the subject level. Data were analyzed using simple hypothesis testing, as well as ordinal regression. *Results*. The 12-year old children had significantly much higher mean PI, CAI, and GI (*P* < 0.001) with 78.6% presenting with gingivitis and 47.8% with moderate gingivitis. In contrast, the figures were 27.2% and 3.1% in the younger group (*P* < 0.001). There were significant variations according to gender, area of residence, and governorate. Regression analysis revealed that mean PI (OR = 35), mean CAI (OR = 7.7), male gender (OR = 1.6), living in rural areas (OR = 1.4), and being from Hajjah or Sana'a were independent risk factors of gingivitis severity in the older group. For the 5-year olds, the determinants were mean PI (OR = 122), male gender (OR = 1.4), and living in Sana'a or Taiz. *Conclusions*. Bad oral hygiene and moderate gingivitis are highly prevalent among Yemeni preschool and school children. Geographical location appeared as important independent risk factors of gingival inflammation.

## 1. Introduction

Chronic gingivitis, a nonspecific inflammatory reaction to dental biofilm bacterial challenge, is the most common oral health problem worldwide in both adults and children. While the disease is largely reversible in nature, it can develop in susceptible hosts into periodontitis, which is characterized by irreversible loss of periodontal attachment [1]. Periodontitis is common in adults, but is still seen in children either as a rare but severely destructive form called aggressive periodontitis or a more common milder form called chronic periodontitis. In fact, high prevalence of these types of periodontitis in children has been reported from some parts of the world [2]. Therefore, early intervention to improve oral hygiene and reduce gingivitis is probably an important approach to prevent periodontitis in children, as is the case with adults [3].

Epidemiological data on gingivitis in children are important for understanding the natural course of the disease, identifying its risk factors, and predicting its time trends [1, 4]. They are also of paramount importance for developing and, later, assessing community preventive programs. However, while overwhelming amount of such data is available for children in developed countries [2, 5], little is known about children in developing countries, although sporadic reports suggest that poor oral hygiene and gingival inflammation are highly prevalent [6–12]. Therefore, the need for national oral health surveys to provide baseline as well as follow-up data in the developing cannot be overemphasized.

Yemen is a poor developing country located South-West of Arabian Peninsula to Kingdom of Saudi Arabia. The country has twenty governorates distributed across four geographical zones that significantly vary with respect to cultural practices, social structure, and livelihood: the highland zone, the western coastal zone (Tehama), the southern coastal zone, and the eastern plateau [13]. Around 35% of the population lives under the poverty line with majority of the poor living in rural areas. However, the prevalence of poverty varies significantly across the geographical zones and among the governorates [14].

Yemen has a total population of around 23 million with children under the age of 15 years making up 43%. However, these do not have access to primary dental healthcare and are not being targeted by any dental educational/preventive programs. Baseline data on oral health status itself are sparse. In a previous study limited to Sana'a city, the capital of the country, we assessed plaque and calculus accumulation and gingival inflammation in a sample of 1489, 6–14-year-old children, revealing gingivitis in 100% of the subjects [6]. Information from other parts of the country is lacking. The purpose of the current study, therefore, was to carry a larger-scale survey to assess the gingival health status in primary school and kindergarten children from several Yemeni cities. 

## 2. Methods

### 2.1. Ethical Consideration

This study was approved by the Ethical Committee of the Deanship of Scientific Research at Khartoum University. Permission to carry on the study was obtained from the Ministry of Education in Yemen as well as authorities of each of the sampled schools. In addition, informed consent was obtained from parents for kindergarten children.

### 2.2. Sampling Areas and Time Period

The study was conducted during the period between April 2003 and October 2005 in five Yemeni governorates: Sana'a, Taiz, Hodeida, Hadramaut, and Hajjah. These were nonrandomly selected to represent the country's geographical zones and according to population density. The five governorates are heterogeneous with respect to culture, social structure, and livelihood [13] as well as severity of poverty. Using the poverty gap index as a measure of the latter, Hajjah is considered the poorest followed by Taiz, Hodeida, Hadramaut, and finally Sana'a [14].

The population of the five selected governorates represents around 50% the total population of Yemen; they contain around 50% of the 12-year-old (sixth grade) school children and about 90% of the 5-year-old private kindergarten children [15].

### 2.3. Study Population and Sampling Procedure

Aiming at a sample size of at least 5000 subjects as recommended by WHO's World Health Survey (WHS) [16], the study population was recruited using stratified cluster random sampling with schools as the primary sampling units or clusters. Following WHS guidelines for cluster size (a maximum of 50 recommended) and based on a pilot study to determine the average number of children that could be examined in one day, a cluster size of 38 subjects was determined. Public primary schools in each governorate were stratified by area of residence (urban, periurban, and rural) and gender, while kindergartens were stratified by gender only since they were exclusively present in urban areas. Schools/kindergartens were then randomly selected in the different strata with the number of clusters roughly proportional to the size of target population in each stratum. However, the number had to be disproportionate for kindergarten children because the size of the target population was exceedingly small (only 2644).

 No 5-year-old children were included from Hajjah, because there were no kindergartens in the whole governorate. Children were randomly selected from one class in each school (grade six for the 12-year olds). Age was confirmed by checking school records. Eventually, a total of 5396 children (4104 of 12 years and 1292 of 5 years) were recruited from 105 public primary schools and 52 private kindergartens. Their distribution by governorate, age group, gender, and area of residence is shown in Table 1. 

### 2.4. Exclusion Criteria and Clinical Examination

Subjects with ongoing or previous orthodontic treatment, under current or previous periodontal treatment, or with history of diabetes, any syndrome, immunosuppression, or intake of immunosuppressive medications were excluded from the study.

Clinically, each child's periodontal status was assessed using the plaque index (PI) according to Silness and Loe [17], the calculus index (CAI) according to Ramfjord [18], and the gingival index (GI) described by Loe and Silness [19], on the six Ramfjord teeth. The mean GI was used to determine categorical gingival status according to Loe [20] as follows: 0, healthy; 0.1–1, mild gingivitis; 1.1–2, moderate gingivitis; 2.1–3, severe gingivitis. All examinations were performed by a single precalibrated examiner. 

### 2.5. Examiner Before-Calibration

The examiner performed measurements of the clinical parameters (PI, CAI, and GI) for 20 subjects on 2 occasions two weeks apart. Intraexaminer variation (differences in mean PI, CAI, and GI between the two occasions) was assessed using paired *t*-test. The examiner was considered calibrated when all differences were not statistically significant differences (*P* > 0.05).

### 2.6. Statistical Analysis

Data were summarized as means ± SD or percentages as appropriate. Significance of differences in clinical parameters by governorate, age group, gender, and area of residence was sought using independent Student's *t*-test or ANOVA for continuous variables (PI, CAI, and GI) and Mann-Whitney and Kruskal-Wallis tests for ordinal variables (gingival status). When necessary, Bonferroni's post hoc multiple comparisons were performed. To identify determinants of  *gingival status* adjusted for confounding in each age group, ordinal regression analysis was carried out using gender, area of residence, mean PI, and mean CAI as factor or covariates as appropriate. A *P* value of ≤0.05 was considered as significant. All analyses were performed using SPSS version 18. 

## 3. Results

### 3.1. Oral Hygiene Status

The PI and CAI scores, stratified by relevant factors for each age group separately, are shown in Tables 2 and 3. The 5-year olds had significantly much lower mean PI and CAI scores than did the 12-year olds (*P* < 0.001). Significantly higher means of both indices in the males compared to the females were observed in the 12- but not the 5-year olds. For the 12-year-old children, those living in the rural areas had the highest PI and CAI scores followed by those in the urban and finally the periurban areas; however, the differences between the rural and urban areas were not statistically significant.

Governorate-wise, the 12-year olds of Hajjah had the highest PI scores followed by those of Sana'a and Hadramaut, then Hodeida, finally Taiz. CAI, however, showed the highest mean in Hadramaut followed by Hodeida, then Hajjah and Sana'a, and finally Taiz. With the exclusion of Hajjah, similar pattern was seen for the 5-year old children with respect to the mean PI, but there were no significant differences in CAI.

### 3.2. Gingival Condition

The mean GI and categorical gingival status are presented in Tables 2 and 4, respectively, for the 5-year-old group and in Tables 3 and 5, respectively, for the older age group. About 27% of the 5-year olds and 78.6% of the 12-year olds had gingivitis. The latter had significantly higher mean GI, with 47.8% demonstrating moderate gingival inflammation compared to only 3.1% of the 5-year olds. Males, in the 12- but not the 5-year-old age group, demonstrated higher mean GI scores compared to females. The 12-year children residing in urban and rural areas had significantly more severe gingival inflammation than did those in periurban areas. 

The 12-year olds from Hajjah and Sana'a demonstrated the highest GI means and rates of moderate gingivitis (around 90%). In comparison, the rates did not exceed 11% in other governorates. In the 5-year olds, there were significant differences among the governorates in mean GI, with Sana'a ranking first and Taiz last; however, moderate gingivitis was observed only in Sana'a (in 9%).

### 3.3. Multivariate Analysis

In the 12-year-old children, independent risk factors of gingivitis severity were mean PI (OR = 35; CI: 27–45), mean CAI (OR = 7.7; CI: 4.4–13.8), male gender (OR = 1.6; CI: 1.3–1.9), living in rural areas (OR = 1.4; CI: 1.1–1.7), and being from Hajjah (OR = 589; CI: 338–668) or Sana'a (OR = 493; CI: 381–875) in comparison with being from Hadramaut. For the 5-year olds, the determinants were mean PI (OR = 122; CI: 76–208), the male gender (OR = 1.4; CI: 1.0–2.0), and living in Sana'a (OR = 47; CI: 25–85) or Taiz (OR = 17; CI: 8.3–34) in contrast with being from Hadramaut.

## 4. Discussion

To the best of our knowledge, this is probably the first country-wide survey to assess oral hygiene and gingival health status among Yemeni preschool and school children. The work was driven by the need for baseline data that highlight the magnitude of the problem and can be used to mobilize authorities towards introducing primary dental healthcare service for children across the country. The indices used are simple, non-time consuming, and easy to use and have proven validity [21]. A sufficiently large representative sample, stratified by gender and level of urbanization, was recruited from 5 governorates taking into consideration variation in population density and heterogeneity with respect to geography, topography, social structure, livelihood, and severity of poverty. However, the study does have a number of limitations. First, children out of kindergarten or school were not included so the findings may not be generalized with confidence to all 5-year and 12-year Yemeni children. Second, sample collection was performed over a long period of time which may have influenced reproducibility of recordings by the examiner. Finally, the authors missed the opportunity to examine other periodontal parameters, such as pocket depth and attachment loss.

The PI mean was 0.34 for the 5-year-old children and 1.12 for the 12-year-old children. The latter is comparable to findings from a previous study conducted on 6–14-year children in Sana'a [8]. Higher or lower figures have been reported from other parts of the world [6, 9, 22–24]. These variations may be attributed to differences in methodology or age of study samples and may also reflect genuine differences in oral hygiene practices, culture, and food habits. The higher plaque index mean in the older group is in line with the majority of previous reports, although few authors did demonstrate an opposite scenario [22, 23]. Males in the 12-year-old group had higher PI means than females which is also consistent with previous reports [9, 25]. This has been attributed to the better tooth brushing behavior in females. However Al-Jasser et al. [26] and Mascarenhas [24] showed opposite findings. School children in the periurban areas showed significantly lower PI mean than school children in rural areas which is in agreement with others [27, 28]; contradictory however was that PI mean in urban areas was comparable to that of rural one, which is very hard to explain. There were also significant differences among the governorates which probably reflect differences in oral hygiene practices or food habits. As expected, differences in CI followed the same pattern as for PI except by governorate, where children from Hadramaut and Hodeida had more calculus than children in other governorates, which may be explained, at least in part, by ethnic variation in calculus formation [29]. 

Plaque-induced gingivitis is almost a universal finding in children. It begins with primary dentition and reaches a pick around puberty [1]. This explains the significant difference in gingival inflammation between the younger and older age groups in the current study. Following differences in PI, males and those living in rural areas had worse gingival condition. In a previous study in Sana'a, we showed that 100% of 6–14-year-old children had some degree of gingivitis [6]; this is somewhat consistent with findings for the 12-year olds from Sana'a in this study where 90% had gingivitis. However, there were significant variations among the governorates in the prevalence and severity of gingivitis which are in harmony with variations in figures reported from other parts of the world [26, 27, 30–33]. These variations may be a reflection of differences in plaque and calculus levels, oral hygiene practices, efficiency of educational programs, food habits, and age groups examined as well as genetic predisposition to gingival inflammation [34]. 

Indeed, the regression analysis in the current study demonstrates clearly that in addition to plaque and calculus accumulation, gender, and level of urbanization as classical risk factors of gingivitis, geographical location also acted as an independent determinant, which provides some sort of evidence for ethnic and possibly genetic variations. Prevalence/severity of poverty did not seem to account for any of the observed intergovernorate variation.

## 5. Conclusions 

Overall, Yemeni preschool and school children suffer from bad oral hygiene and high prevalence of moderate gingivitis, particularly in the older age group. Certain geographical areas of high risk were identified. These findings should drive future research and be used as a basis for planning national preventive program. 

## Figures and Tables

**Table 1:** Distribution of the sample by governorate, age, gender, and area of residence.

	Governorate				
	Sana'a *N* (%)	Taiz *N* (%)	Hodeida *N* (%)	Hajjah *N* (%)	Hadramaut *N* (%)
Age					
5 years (*n* = 1292)	456 (35.3)	456 (35.3)	152 (11.8)	000 (00.0)	228 (17.6)
12 years (*n* = 4104)	1216 (29.6)	836 (20.4)	684 (16.7)	684 (16.7)	684 (16.7)
Total (*n* = 5396)	**1672 (31.0)**	**1292 (23.9)**	**836 (15.5)**	**684 (12.7)**	**912 (16.9)**
Gender					
Male (*n* = 2698)	836 (31.0)	646 (23.9)	418 (15.5)	342 (12.7)	456 (16.9)
Female (*n* = 2698)	836 (31.0)	646 (23.9)	418 (15.5)	342 (12.7)	456 (16.9)
Total (*n* = 5396)	**1672 (31.0)**	**1292 (23.9)**	**836 (15.5)**	**684 (12.7)**	**912 (16.9)**
Area of residence (for 12 years)					
Urban (*n* = 1520)	608 (40.0)	228 (15.0)	228 (15.0)	228 (15.0)	228 (15.0)
Rural (*n* = 1292)	304 (23.5)	304 (23.5)	228 (17.6)	228 (17.6)	228 (17.6)
Periurban (*n* = 1292)	304 (23.5)	304 (23.5)	228 (17.6)	228 (17.6)	228 (17.6)
Total (*n* = 4104)	**1216 (29.6)**	**836 (20.4)**	**684 (16.7)**	**684 (16.7)**	**684 (16.7)**

**Table 2:** Mean ± SD plaque, gingival, and calculus indices for the 5-year-old age group by gender and governorate.

	Plaque index	Calculus index	Gingival index
Gender			
Male (*n* = 646)	0.34 ± 0.46	0.00 ± 0.00	0.22 ± 0.02
Female (*n* = 646)	0.35 ± 0.48	0.00 ± 0.01	0.17 ± 0.01
Total (*n* = 1292)	**0.35** **± 0.47**	**0.00 ± 0.01**	**0.19 ± 0.38**
* t* *-test *	*0.77 *	*0.037 *	*0.32 *
Governorate			
Sana'a (*n* = 456)	0.51 ± 0.47	0.00 ± 0.10	0.39 ± 0.49
Taiz (*n* = 456)	0.11 ± 0.28	0.00 ± 0.00	0.06 ± 0.20
Hodeida (*n* = 152)	0.35 ± 0.50	0.00 ± 0.00	0.11 ± 0.28
Hadramaut (*n* = 228)	0.50 ± 0.57	0.00 ± 0.00	0.13 ± 0.33
Total (*n* = 1292)	**0.35 ± 0.47**	**0.00** **± 0.01**	**0.19 ± 0.38**
* ANOVA *	*<0.001 *	*0.61 *	*<0.001 *

**Table 3:** Mean ± SD plaque, gingival, and calculus indices for the 12-year-old age group by gender, area of residence, and governorate.

	Plaque index	Calculus index	Gingival index
Gender			
Male (*n* = 2052)	1.17 ± 0.60	0.09 ± 0.18	1.03 ± 0.64
Female (*n* = 2052)	1.06 ± 0.49	0.04 ± 0.13	0.91 ± 0.62
Total (*n* = 4104)	**1.12 ± 0.55**	**0.07 ± 0.16**	**0.97 ± 0.64**
* t* *-test *	*<0.001 *	*<0.001 *	*<0.001 *
Area of residence (for 12 years)			
Urban (*n* = 1520)	1.12 ± 0.53	0.07 ± 0.19	1.01 ± 0.56
Rural (*n* = 1292)	1.19 ± 0.54	0.07 ± 0.15	1.02 ± 0.69
Periurban (*n* = 1292)	1.04 ± 0.57	0.05 ± 0.14	0.88 ± 0.65
Total (*n* = 4104)	**1.12 ± 0.55**	**0.07 ± 0.16**	**0.97 ± 0.64**
* ANOVA *	*<0.001 *	*0.005 *	*<0.001 *
Governorate			
Sana'a (*n* = 1216)	1.26 ± 0.49	0.03 ± 0.12	1.38 ± 0.31
Taiz (*n* = 836)	0.71 ± 0.56	0.02 ± 0.08	0.54 ± 0.59
Hodeida (*n* = 684)	1.01 ± 0.67	0.10 ± 0.20	0.61 ± 0.63
Hajjah (*n* = 684)	1.33 ± 0.34	0.04 ± 0.11	1.43 ± 0.31
Hadramaut (*n* = 684)	1.25 ± 0.69	0.16 ± 0.25	0.69 ± 0.63
Total (*n* = 4104)	**1.12** **± 0.55**	**0.049** **± 0.14**	**0.97** **± 0.64**
* ANOVA *	*<0.001 *	*<0.001 *	*<0.001 *

**Table 4:** Level of gingival inflammation in the 5-year olds by gender and governorate.

	None *N* (%)	Mild *N* (%)	Moderate *N* (%)	Severe *N* (%)
Gender				
Male (*n* = 646)	455 (70.4)	173 (26.8)	18 (2.8)	0 (0.0%)
Female (*n* = 646)	485 (75.1)	139 (22.5)	22 (3.4)	0 (0.0%)
Total (*n* = 1292)	**940 (72.8)**	**312 (24.1)**	**40 (3.1)**	**0 (0.0%)**
* Mann-Whitney *	*0.08 *			
Governorate				
Sana'a (*n* = 456)	221 (48.5)	195 (42.8)	40 (8.8)	0 (0.0%)
Taiz (*n* = 456)	400 (87.7)	56 (12.3)	0 (0.0)	0 (0.0%)
Hodeida (*n* = 152)	126 (82.9)	26 (17.1)	0 (0.0)	0 (0.0%)
Hadramaut (*n* = 228)	193 (84.6)	35 (15.4)	0 (0.0)	0 (0.0%)
Total (*n* = 1292)	**940 (72.8)**	**312 (24.1)**	**40 (3.1)**	**0 (0.0%)**
* Kruskal-Wallis *	*<0.001 *			

**Table 5:** Level of gingival inflammation in the 12-year olds by gender, area of residence, and governorate.

	None *N* (%)	Mild *N* (%)	Moderate *N* (%)	Severe *N* (%)
Gender				
Male (*n* = 2052)	367 (17.9)	625 (30.5)	1059 (51.6)	1 (0.00%)
Female (*n* = 2052)	513 (25.0)	635 (30.9)	904 (44.1)	0 (0.00%)
Total (*n* = 4104)	**880 (21.4)**	**1260 (30.7)**	**1963 (47.8)**	**1 (0.00%)**
* Mann-Whitney *	*<0.001 *			
Area of residence (for 12 years)				
Urban (*n* = 1520)	239 (15.7)	487 (32.0)	794 (52.2)	0 (0.00%)
Rural (*n* = 1292)	301 (23.3)	349 (27.0)	641 (49.9)	1 (0.00%)
Periurban (*n* = 1292)	340 (26.3)	424 (32.8)	528 (40.9)	0 (0.00%)
Total (*n* = 4104)	**880 (21.4)**	**1260 (30.7)**	**1963 (47.8)**	**1 (0.00%)**
* Kruskal-Wallis *	*<0.001 *			
Governorate				
Sana'a (*n* = 1216)	0 (0.0)	129 (10.6)	1086 (89.3)	1 (0.01%)
Taiz (*n* = 836)	392 (46.9)	351 (42.0)	93 (11.1)	0 (0.0%)
Hodeida (*n* = 684)	269 (39.3)	339 (49.6)	76 (11.1)	0 (0.0%)
Hajjah (*n* = 684)	0 (0.0)	52 (7.6)	632 (92.4)	0 (0.0%)
Hadramaut (*n* = 684)	219 (32.0)	389 (56.9)	76 (11.1)	0 (0.0%)
Total (*n* = 4104)	**0880 (21.4)**	**1260 (30.7)**	**1963 (47.8)**	**1 (0.0%)**
* Kruskal-Wallis *	*<0.001 *			
